# The nurse in the context of chronic disease

**DOI:** 10.1590/0104-1169.0000.2517

**Published:** 2015

**Authors:** Maria Cecilia Bueno Jayme Gallani

**Affiliations:** Maria Cecilia Bueno Jayme Gallani is Associate Editor of the Revista Latino-Americana de Enfermagem and Full Professor of the Faculté des Sciences Infirmières, Université Laval, Québec, Canada. E-mail: maria-cecilia.gallani@fsi.ulaval.ca


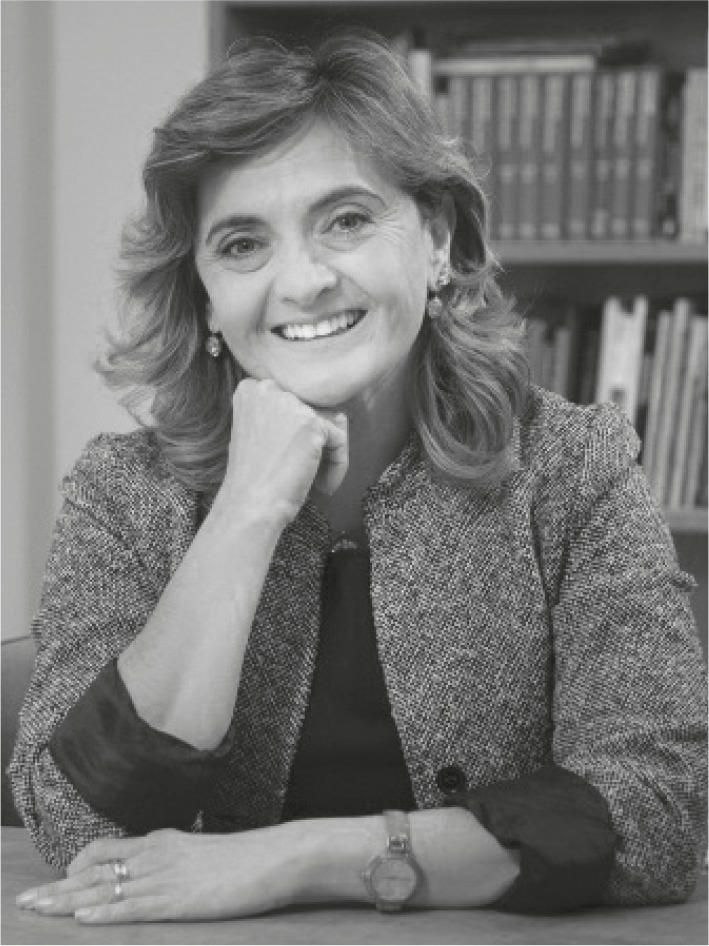


A previous RLAE^(^ 1 ^)^ editorial highlights, very appropriately, the importance of producing knowledge in the area of chronic disease. The literature, as well as official documents from the various health care bodies, confirm that this is a very pertinent concern. The scale of non-communicable diseases continues to grow, causing world leaders to place such diseases on their development agendas, recognizing the great threat they pose to health, economies and societies^(^ 2 ^)^.

The growth of chronic disease poses many challenges. The first relates to the heterogeneity of epidemiological transition observed in different populations. Beyond differences observed in the profile of risk factors^(^ 3 ^)^, many low and middle income countries experience the coexistence of the consequences of the global phenomena of ageing populations/globalization/urbanization as well as the impact of communicable diseases, which remain high. The projected trends provoke serious concern, especially in populations which, although greatly affected, have fewer resources at their disposal to tackle the problem^(^ 2 ^)^.

Another challenge concerns the lengthy duration of the natural course, as well as its complexity and the irregular trajectory of evolution of chronic diseases, which rarely occur in isolation. This particular pattern necessarily imposes a paradigm of continuity of care and of health care services, as well as establishing complex interventions, but proving difficult to effect in practice. The accessibility of health care and services is still limited, as is establishing continuity and standardizing care. Also important is the evident lack of integration of interventions, the efficiency and efficacy of which have been proved, not only in controlling disease and limiting functional incapacity, but rather in promoting quality of life.

This editorial aims to draw attention to the importance of the great contribution nurses make in optimizing the quality of health care services, in the context of chronic diseases. In fact, it could be said that the nurse is one of the health care professionals, if not THE health care professional, who is most involved in this context in the most diverse of cultures, as: 1) nursing is the largest workforce in health care, being on the front line of direct contact with patient-family-community; 2) the nurse is, par excellence, the professional trained to ensure continuity of care aimed at an appropriate trajectory through all health sectors - from primary to quaternary and vice versa; and 3) the nurse is trained to deal with all the spheres making up wellbeing on the health care spectrum: physical/clinical, emotional, social, cognitive and spiritual aspects. Considering the singularity of the individual's experience throughout the health-disease process and, simultaneously, their integration into society. 

The nurse's contribution in the context of chronic diseases depends primarily on solid training that encourages the exercise of consistent, profound and wide ranging clinical judgment. It also depends on the capacity to propose and evaluate innovative interventions, aiming to prevent or stabilize chronic diseases. But in order for this to be possible, however, research into nursing interventions based on theoretical-methodological needs to increase, allowing the most efficient and efficacious to be identified, considering the evaluation of quality of life as the outcome. The research into interventions in nursing need to evolve in complexity and coverage, aiming for an increasingly broad approach towards the individual as a whole, not fragmented into their different comorbidities and risk factors. Research along the whole health care spectrum is also essential, considering the complex periods of transition marking the evolution of the patient living with chronic disease.

Finally, dialogue between research and practice is indispensable, it enables nursing research results to be translated and established as effective clinical practice. The only way that knowledge can be transferred into practice is solid partnership between practicing nurses, operating in clinical practice and in different levels of management, and the academic world. It is essential that the nurse exercises leadership within the different domains in which they operate^(^ 4 ^)^, based on negotiation abilities and, above all, on knowledge.

 In conclusion, the challenges faced by nurses in the XXI century, in approaching chronic diseases, indicate the need for a new care paradigm, of research into intervention and intra-professional relationships.
